# Depressive symptoms and quality of life prior to metabolic surgery in Cape Town, South Africa

**DOI:** 10.4102/sajpsychiatry.v28i0.1783

**Published:** 2022-09-30

**Authors:** Wilma M. Kruger-Steyn, Jeanne Lubbe, Kerry-Ann Louw, Laila Asmal

**Affiliations:** 1 Department of Psychiatry, Faculty of Medicine and Health Sciences, Stellenbosch University, Cape Town, South Africa; 2 Division of Surgery, Faculty of Medicine and Health Sciences, Stellenbosch University, Cape Town, South Africa

**Keywords:** obesity, depression, metabolic surgery, quality of life, back pain

## Abstract

**Background:**

Depression has been shown to have a negative impact on the outcomes of metabolic surgery and quality of life (QOL). Currently, there are limited data on mental distress and QOL in metabolic surgery candidates in South Africa.

**Aim:**

This study aimed to determine the prevalence of depressive symptoms at the time of presurgical assessment in participants undergoing metabolic surgery.

**Setting:**

The Obesity and Metabolic Surgery Initiative at Tygerberg Hospital.

**Methods:**

We conducted pre-operatively a retrospective cross-sectional study on patients who underwent metabolic surgery from September 2017 to September 2019. Participants were profiled in terms of metabolic parameters, depressive symptoms and QOL.

**Results:**

Of the 157 participants assessed, 88% were female with a body mass index in the super obese range. Twenty-two percent of participants had depressive symptoms. Metabolic surgery candidates with depressive symptoms had a significantly poorer overall QOL score compared with those without depressive symptoms. When controlling for all other variables, an increase in QOL score was shown to decrease the odds of current depressive symptoms, whilst back pain on non-narcotic medication and having had a stroke were found to increase the odds of current depressive symptoms.

**Conclusion:**

This study highlights the complex interplay between metabolic, clinical and psychiatric factors in patients undergoing metabolic surgery. The study highlights the vital role of a psychiatrist as part of a multidisciplinary team pre- and post-operatively in the early identification of depressive symptoms. Psychiatrists may have an important role to play as part of the multidisciplinary team in metabolic surgery, including screening for mental health problems pre- and post-operatively, providing psychoeducation and relevant pharmacological treatment and psychotherapy where needed.

**Contribution:**

This study expands our limited knowledge of psychiatric comorbidity (in particular depressive symptoms and associated factors) in people undergoing metabolic surgery in low- and middle-income countries.

## Introduction

Given the steady increase of obesity rates worldwide and the associated health risks of a high body mass index (BMI), obesity has become a major global health problem.^1^ Almost two in three of the world’s obese reside in low- to middle-income countries (LMICs).^1^ According to a 2021 South African study, 38.5% and 11.9% of adult women and men, respectively, were obese.^2^ A multitude of chronic illnesses are associated with a high BMI, including dyslipidaemia, hypertension, stroke, insulin resistance, type 2 diabetes mellitus (T2DM) and pulmonary diseases such as obstructive sleep apnoea.^3^ Excess weight impacts on the musculoskeletal system and various musculoskeletal disorders are also known to cause morbidity in this population.^3^ People who are obese are also more likely to have comorbid psychiatric conditions.^3^

There appears to be a bidirectional association between obesity and mental health disorders. There is existing evidence that levels of obesity are higher in patients with depression than in the general population.^4^ Luppino et al.^5^ reported on the relationship between being overweight (BMI 25 kg/m² – 29.9 kg/m²) or obese (BMI > 30 kg/m²) and depression, where the lifetime risk of an obese patient developing depression was 55%, and the risk of a depressed patient becoming obese was increased by 58%. The mechanisms for the bidirectional association between depression and obesity are complex, with numerous biological and psychological pathways implicated.^5^ Obesity is a pro-inflammatory state, with adipocytes synthesising adipokines and hormones, leading to low-grade systemic inflammation in some obese patients.^3^ Furthermore, it has been demonstrated that depressive disorders are associated with inflammatory states, with increased levels of C-reactive proteins, tumour necrosis factor-alpha (TNF-alpha) and interleukin-6 (IL-6) in patients with depression versus healthy controls.^6^ Another proposed mechanism accounting for the bidirectional association between obesity and depression is the hypothalamic-pituitary-adrenal axis and its dysregulation.^5^

Given the immense burden on health care services, there is a need for suitable treatments for patients with obesity and its associated comorbidities. When compared with other interventions, metabolic surgery has superior outcomes.^7^ However, even though superior outcomes have been described in bariatric patients, the risk of suicide and unsuccessful weight loss in some bariatric surgery patients make it desirable to employ a pre-operative objective psychiatric assessment with standardised instruments, post-op monitoring and effective psychological interventions.^8,9,10^ It has been shown that a reduction in weight through metabolic surgery leads to a reduction in depressive symptoms in patients through various biological and psychosocial pathways.^11^ However, metabolic surgery has only been performed to a limited extent in state facilities in South Africa, and as such, not much is known about the demographic, metabolic and clinical profiles of patients undergoing metabolic surgery in this sector.^12^ Past research indicates that metabolic surgery candidates have a high comorbidity of mental health problems, notably depression and anxiety.^11,13^ It has been shown that 20% – 50% of metabolic surgery candidates in the United States of America (USA) have a history of mood disorders, with depression being the most common.^14^ Depression is also more common in candidates for metabolic surgery than in the general population.^14^ Furthermore, metabolic surgery candidates have poorer health-related quality of life (QOL) scores when compared with the general population.^13^ Quality of life refers to an individual’s perception of well-being in the domains of social, occupational, economic, physical and mental health.^14^ Health-related QOL measures the impact of an illness and its treatment on a patient in these domains.^15^ Obesity impacts QOL measures, and the impact is more severe at higher BMI levels.^16^

We hypothesised that in participants who undergo metabolic surgery, depressive symptoms are associated with a poorer QOL and metabolic profile prior to surgery. Because of the limited data available on metabolic surgery and psychiatric comorbidities in LMICs, we believed a study in this specific setting would be a valuable contribution to the current body of knowledge in this field. The aim of this study was to determine the prevalence of depressive symptoms at the time of presurgical assessment in a sample of participants undergoing metabolic surgery. We further aimed to describe the demographic characteristics, clinical and metabolic profiles and the QOL of participants who screened positive for depressive symptoms compared with participants who screened negative.

## Methods

### Study design

We conducted a descriptive, cross-sectional, retrospective analysis of prospectively collected pre-operative data from September 2017 to September 2019. This study is a secondary analysis of data obtained from the parent study, an observational prospective descriptive study of the feasibility, safety and efficacy outcomes of consecutive patients undergoing metabolic surgery at the Obesity and Metabolic Surgery Initiative at Tygerberg Hospital (OMIT). The safety and efficacy findings have been published elsewhere.^12^ Each participant in the parent study underwent a comprehensive pre-operative assessment by the bariatric surgeon of OMIT.

### Study setting

The parent study was conducted at Tygerberg Hospital, located in Parow, Cape Town, South Africa. Tygerberg Hospital, the largest hospital in the Western Cape, is a tertiary state hospital that predominantly serves a population of low- to middle-income patients. The hospital is a teaching facility of the Faculty of Medicine and Health Sciences, Stellenbosch University. Tygerberg Hospital is the only academic hospital in South Africa providing the service of metabolic surgery and thus receives referrals from all over the country, including the private sector.

### Participant selection

We included all 157 participants who met the inclusion and exclusion criteria of the parent study. They were all voluntary patients and provided written, informed consent to participate in the study. In brief, participants were considered for the parent study according to guideline recommendations for metabolic surgery.^17^ All adult patients between the ages of 20 and 60 years with morbid (BMI ≥ 40 kg/m^2^) or severe obesity (BMI ≥ 35 kg/m^2^) and with the comorbid obesity-related disease were included in the study. Patients who were assessed as having too high an anaesthetic or surgical risk, those having had a current or previous history of illicit substance use or excessive alcohol use, those with an inability to commit to follow-up care, and pregnant or breastfeeding patients (or planning pregnancy in the next two years) were excluded from the study. Poorly controlled psychiatric illness was an exclusion criterion of the parent study; however, no patients required exclusion on these grounds.

### Data collection and measures

Each participant underwent presurgical assessment by the metabolic surgeon at nonuniform time points prior to their metabolic surgery. All participants were asked about current depressive symptoms using a screening questionnaire designed specifically for the OMIT study. Assessment of depressive symptoms was based on the clinical assessment of the attending clinician, who asked about the presence of symptoms of depression including low mood, anhedonia, fatigue, poor sleep and feelings of worthlessness. No specific rating scale or formal *Diagnostic and Statistical Manuel of Mental Disorders, Fifth Edition* (*DSM-5*) criteria were used to diagnose depressive symptoms. Based on patients’ self-report of their depressive symptoms and required treatment, the attending clinician categorised patients as no current symptoms, mild symptoms not requiring medication or therapy, moderate symptoms accompanied by some impairment possibly requiring medication or therapy, moderate symptoms accompanied by significant impairment requiring medication or therapy and severe depression requiring intensive treatment, including hospitalisation. For this study, a participant was identified as having depressive symptoms if they screened positive for any current depressive symptoms at least one being low mood or anhedonia (mild and above). All participants requiring psychiatric treatment were referred to the collaborating Tygerberg Hospital Eating Disorders Unit for further care.

Metabolic parameters were measured for each participant. Type 2 diabetes mellitus was defined as a known diagnosis of T2DM on treatment or a serum HbA1c > 6.5% or two fasting glucose tests > 125 mg/dL. Prediabetes was defined as a fasting blood glucose between 100 mg/dL and 125 mg/dL. Height was measured in centimetres, and weight was measured in kilograms. Body mass index is universally used to define obesity and calculated from weight in kilograms and height in meters as kg/m^2^.^18^ Blood pressure, fasting blood glucose and a lipogram were also measured for each participant. Borderline hypertension was defined as a systolic reading of 120 mmHg – 139 mmHg and normal diastolic blood pressure; Stage 1 hypertension was defined as a blood pressure of 140 mmHg–159 mmHg/90 mmHg–99 mmHg or controlled on one medication, and Stage 2 was defined as >160 mmHg/>100 mmHg or controlled on multiple medications. Borderline dyslipidaemia was defined as LDL cholesterol 3.4 mmol/L – 4.1 mmol/L, total cholesterol 5.2 mmol/L – 6.2 mmol/L and triglycerides 1.7 mmol/L – 2.3 mmol/L. Confirmed dyslipidaemia was defined as LDL cholesterol > 4.1 mmol/L, total cholesterol > 6.2 mmol/L and triglycerides > 2.3 mmol/L. All participants completed a general medical and study questionnaire, which included sociodemographic details (age and gender) as well as a medical and psychiatric history. Medical comorbidities related to obesity were all screened, including obstructive sleep apnoea, ischaemic heart disease, cerebrovascular accidents or stroke and back pain. We categorised back pain based on whether the participants were on no treatment, narcotic medication or non-narcotic medication. Non-narcotic medication refers to a subclass of analgesics that do not bind to opioid receptors, whereas narcotic medication refers to natural or synthetic derivatives of opium or morphine that bind to opioid receptors to induce analgesia.^19^ A history of smoking, alcohol and illicit substance use was documented based on self-report history.

Quality of life was assessed using the Moorehead-Ardelt QOL Questionnaire II.^20^ This questionnaire is a disease-specific instrument designed to measure self-perceived QOL in the overweight to super obese population seeking medical and surgical intervention. It correlates well with other established health and well-being measures in the field of psychiatry^20^ and has been validated in the USA (Cronbach’s α = 0.84)^20^ and Europe (Cronbach’s α = 0.80 and 0.82)^21,22^ but not in South Africa. Six domains are examined: eating behaviour, self-esteem, physical well-being, work, sexuality and social relationships.^20^ A 10-point Likert scale ranging from −0.50 to +0.50 was used for scoring each domain. In addition to domain scores, a total score was calculated by summing up the six domain scores. The total score was categorised for QOL as very poor (−3.0 to −2.1), poor (−2.0 to −1.1), fair (−1.0 to +1.0), good (+1.1 to +2.0) and very good (+2.1 to +3.0).

### Statistical analyses

Stata version 13 was used to analyse the data.^23^ Descriptive statistics are provided for all demographic and clinical variables. Prevalence of depression and a positive history of depression were described using relative frequency and percentages with 95% confidence intervals. Associations between depression and metabolic parameters, QOL and sociodemographic variables were derived from chi-square test in the case of categorical variables and *t*-test in the case of continuous variables. All factors associated with depression on bivariate analyses at a *p* < 0.1 level were entered into a logistic regression model to ensure that potentially important variables, including confounders as well as within-category group differences, were not missed.^24^ Backward selection procedures were then used to eliminate nonsignificant variables at a *p* < 0.05 level and thereby control for confounding factors. Statistical significance was set at *p* < 0.05 throughout the analyses except for entry into the logistic regression model.

### Ethical considerations

The study received ethical approval from the Health Research Ethics Committee (HREC) of Stellenbosch University (HREC ref. no. S18/01/003). All participants provided written informed consent. The study was conducted in accordance with the South African Good Clinical Practice Guidelines (DOH 2006) and the Declaration of Helsinki (2013).

## Results

A total of 157 participants were assessed at Tygerberg Hospital before undergoing metabolic surgery. The baseline demographic characteristics are presented in Table 1. In this sample, participants were predominantly female (*n* = 138, 88%), with a BMI in the super obese range. Most participants reported no current depressive symptoms (*n* = 122, 77.2%) and no prior history of depression (*n* = 117, 74.5%). Participants largely rated their QOL as ‘fair’ with a mean (SD) QOL score of 3.2 (0.72). Metabolic surgery candidates with depressive symptoms had a significantly poorer overall QOL score (*p* = 0.048) compared with those without depressive symptoms.

**TABLE 1:** Baseline demographic characteristics for participants who screened positive versus those who screened negative for depressive symptoms.

Variables	Total sample (*n* = 157)					Depressive symptoms											
	*n*	%	Mean	SD	Range	Screen positive (*n* = 35, 22%)					Screen negative (*n* = 122, 78%)					*p*	95% CI
						*n*	%	Mean	SD	Range	*n*	%	Mean	SD	Range		
**Age in years**	-	-	43.67	9.44	-	-	-	45.88	9.66	-	-	-	43.03	9.32	-	0.11	−6.41 to 0.70
**Gender**																	
Female	138	88	-	-	-	33	94.3	-	-	-	105	86.1	-	-	-	0.19	−0.18 to 0.016
Male	19	12	-	-	-	2	5.7	-	-	-	17	13.9	-	-	-		
**BMI**	-	-	51.04	8.82	32.16 – 81.32	-	-	49.66	6.10	-	-	-	51.44	9.20	-	0.28	−1.48 to 5.04
**QOL** †	-	-	0.40	1.08	-	-	-	0.07	1.06	-	-	-	0.49	1.08	-	**0.048**	0.004 to 0.83

BMI, body mass index; QOL, quality of life.

† , *n* = 152, 34 patients screened positive for depression, 118 screened negative for depression.

BMI, body mass index; QOL, quality of life.

Metabolic parameters were deranged in most participants: over two-thirds were prediabetic or had T2DM; prehypertension or hypertension was prevalent in most of the cohort (*n* = 139, 88.5%), and only 44 participants (28%) had normal lipid levels (Table 2). Symptoms of obstructive sleep apnoea were experienced by most of the participants (*n* = 145, 92.4%). A total of 100 participants (63.7%) reported symptoms of back pain, but most participants reported no treatment required for their back pain. Ischaemic heart disease was present in 89 participants (56.7%), and five participants (3.2%) had a history of stroke. Metabolic surgery candidates with depressive symptoms did not differ significantly from those without depressive symptoms in terms of BMI, T2DM, hypertension, lipid level or ischaemic heart disease.

**TABLE 2:** Baseline clinical characteristics for participants who screened positive versus those who screened negative for depressive symptoms.

Variables	Total sample (*n* = 157)		Depressive symptoms					
	*n*	%	Screen positive (*n* = 35, 22%)		Screen negative (*n* = 122, 78%)		*p*	95% CI
			*n*	%	*n*	%		
**T2DM**								
None	50	31.90	14	40.0	36	29.50	0.26	−0.50 to 0.14
Prediabetic	43	27.40	7	20.0	36	29.50	-	-
Oral medication	45	28.70	12	34.3	33	27.10	-	-
Insulin alone	2	1.30	0	-	2	1.60	-	-
Oral med and insulin	16	10.20	2	5.7	14	11.50	-	-
Poor control on meds	1	0.64	0	-	1	0.82	-	-
**Hypertension**								
No hypertension	18	11.50	3	8.6	15	12.30	0.43	−0.21 to 0.51
Prehypertension	48	30.60	9	25.7	39	32.00	-	-
Stage 1	44	28.00	11	31.4	33	27.00	-	-
Stage 2	43	27.40	12	34.3	31	25.40	-	-
Poor control on meds	4	2.60	0	-	4	3.30	-	-
**Lipid levels**								
Normal	44	28.00	11	31.4	33	27.00	0.78	−0.40 to 0.53
High, no medication	52	33.10	8	2.9	44	36.10	-	-
High, on medication	61	38.90	16	45.7	45	36.90	-	-
**OSA**								
None	12	7.60	1	2.9	11	9.00	0.06	−0.13 to 2.12
Symptoms	142	90.50	32	91.4	110	90.20	-	-
Confirmed, no device	1	0.64	1	2.9	0	-	-	-
Confirmed, device	2	1.30	1	2.9	1	0.80	-	-
**Back pain**								
None	57	36.30	7	20.0	50	41.00	0.06	−0.01 to 0.84
Symptoms, not requiring treatment	68	43.30	17	48.6	51	41.80	-	-
Symptoms, requiring non-narcotic medication	23	14.70	10	28.6	13	10.70	-	-
Symptoms, requiring narcotic medication	9	5.70	1	2.9	8	6.60	-	-
**IHD**								
Yes	89	56.70	20	57.0	69	56.60	0.95	−0.73 to 0.78
No	68	43.30	15	43.0	53	43.40	-	-
**Stroke** †								
Yes	5	3.20	3	8.6	2	1.70	0.06	−0.16 to 0.02
No	-	-	32	91.4	119	98.30	-	-

OSA, obstructive sleep apnoea; T2DM, type 2 diabetes mellitus; IHD, ischaemic heart disease.

† , *n* = 156, 35 patients screened positive for depression and 121 screened negative for depression.

Substance use disorder and illicit substance use were exclusion criteria for metabolic surgery, and participants of this study used alcohol only occasionally or not at all (*n* = 155, 99%); the remaining two participants reported frequent use of alcohol (Table 3). Most of the participants were nonsmokers (*n* = 134, 85.4%). Metabolic surgery candidates with depressive symptoms did not differ significantly from those without depressive symptoms in terms of alcohol or tobacco use.

**TABLE 3:** Baseline substance use characteristics for participants who screened positive versus those who screened negative for depressive symptoms.

Variables	Total sample (*n* = 157)		Depressive symptoms					
	*n*	%	Screen positive (*n*= 35, 22%)		Screen negative (*n* = 122, 78%)		*p*	95% CI
			*n*	%	*n*	%		
**Alcohol**								
None	71	45	16	45.7	55	45.1	0.899	0.75 – 1.80
Rare	42	27	9	25.7	33	27.1	-	-
Occasional	42	27	9	25.7	33	27.1	-	-
Frequent	2	1	1	2.9	1	0.82	-	-
**Tobacco**								
None	134	85.5	27	77	107	88	0.099	0.96 – 1.88
Rare	1	0.6	0		1	1	-	-
Occasional	4	2.6	1	3	3	3	-	-
Frequent	18	11.5	7	20	11	9	-	-

Based on the results of the above-mentioned bivariate analyses, the following variables qualified for inclusion in the logistic regression model: OSA (*p* = 0.06), previous stroke (*p* = 0.06), back pain (*p* = 0.06) and tobacco use (*p* = 0.099). Adjusted odds ratios and *p*-values illustrating the strength of the association between depression and demographic and clinical variables are presented in Table 4. When controlling for all other variables, an increase in QOL was shown to decrease the odds of depressive symptoms (OR = 0.607 [95% CI, 0.389 – 0.964], *p* = 0.027), whilst back pain on non-narcotic medication (OR = 7.041 [95% CI, 1.936 – 25.61], *p* = 0.003) and having had a stroke (OR = 10.478 [95% CI, 1.024 – 107.223], *p* = 0.048) were found to increase the odds of depressive symptoms.

**TABLE 4:** Association between clinical features, demographic characteristics and depression: multivariate logistic regression model.

Variable	AOR	95% CI	*p*
**OSA**			
Nil	1 (reference)	-	-
Symptoms	1.54	0.17 – 14.33	0.70
Confirmed, no device	†	-	-
Confirmed, device	1.63	0.04 – 63.92	0.79
**Back pain**			
Nil	1 (reference)	-	-
Symptoms, no treatment	2.48	0.85 – 7.20	0.09
Symptoms, on non-narcotic medication	7.04	1.94 – 25.61	**0.003**
Symptoms, on narcotic medication	0.532	0.05 – 6.06	0.611
**Tobacco**			
Nil	1 (reference)	-	-
Rare	†	-	-
Occasional	0.42	0.24 – 7.21	0.550
Frequent	2.87	0.87 – 9.43	0.083
**Stroke**			
No	1 (reference)	-	-
Yes	10.48	1.02 – 107.22	**0.048**
**QOL**	0.61	0.39 – 0.95	**0.027**

AOR, adjusted odds ratio; OSA, obstructive sleep apnoea; QOL, quality of life; BMI, body mass index.

† , Variable omitted to ensure model stability because only a single participant answered.

## Discussion

As far as we are aware, the present study is the first in South Africa to identify the prevalence of and discuss depressive symptoms in metabolic surgery candidates pre-operatively. The first important finding from this study was that most participants had no depressive symptoms at the time of presurgical assessment and fair QOL scores. The second important finding was that those participants with depressive symptoms had significantly lower QOL scores and were more likely to report back pain requiring non-narcotic analgesia and to have had a previous stroke compared with participants without depressive symptoms.

The prevalence of 22% of depressive symptoms in this sample is similar to that found in other studies. In a meta-analysis by Dawes et al.,^14^ the prevalence of depression was 19% in patients seeking and undergoing metabolic surgery. Van der Merwe et al.^25^ reported that 25.5% of males and 53.2% of females had depression prior to metabolic surgery in their South African centre sample of 820 candidates.

In our study, depressive symptoms were associated with lower QOL pre-operatively. Whilst heterogeneity in QOL measures and study design makes a direct comparison between our QOL scores and that of other studies difficult, our findings are consistent with the nature of the association between QOL and depression found in other metabolic surgery studies. Obesity is associated with a decreased health-related QOL for patients in the physical, social and psychological domains.^19^ Metabolic surgery has been associated with long-term reductions in depressive symptoms among individuals postoperatively,^11,14^ and together with long-term weight loss post surgery, has been shown to have a positive effect on health-related QOL in severely obese individuals.^16^ This would be an area for further study in this population.

In our sample, almost two thirds of patients with depressive symptoms reported chronic back pain. The bidirectional relationship between depression and chronic pain is well described and may be because of shared neural mechanisms.^26^ Depression may cause increased perceived chronic pain, while chronic pain may also cause depression.^27^ In a large Canadian population-based study, participants with chronic back pain were more likely to be depressed than participants without pain.^28^ Furthermore, mood disorders are associated with higher levels of perceived chronic pain.^27^ Studies have also shown that pain-free participants with depression are more likely to develop neck or back pain at 6 and 12 months of follow-up than participants without depression.^29^ There is evidence that inflammation plays a role in the aetiology of depression, and many studies have looked at the potential of anti-inflammatory agents in the treatment of depression.^30^ Opioid use increases the risk for depression,^31^ and it would be expected that the group of patients with back pain on narcotic medication would have a stronger association with depression. However, in our study, the association was found between depressive symptoms and back pain requiring non-narcotic medication. Possible explanations for this may be that the pain of participants on non-narcotic treatment was less optimally treated, resulting in a greater degree of back pain and subsequent depressive symptoms than the group taking narcotic treatment.

In our study, there was a weak association between depressive symptoms pre-operatively and history of stroke. According to the literature, there is a bidirectional association between depression and stroke, where depression is a known independent risk factor for stroke as well as being one of the most common neuropsychiatric sequelae following a cerebrovascular incident.^32^ A meta-analysis found that 31% of stroke survivors developed depression at any time point in the 5 years following stroke,^33^ where symptoms of depression most frequently occurred during the first year following a stroke.^34^ In the Swedish Obese Subjects (SOS) intervention study, 0.7% of participants had a previous stroke,^16^ whereas 3.2% of participants in our study had a history of stroke. Van der Merwe et al.^25^ found that 2% of males and 2.8% of females had a history of minor cerebrovascular incidents and transient ischaemic attacks prior to metabolic surgery in their sample of 820 candidates. According to a study by Bertram et al.,^35^ the incidence of stroke per 100 000 population in South Africa in 2008 was 465 for men and 615 for women. The fact that participants in our study had an average BMI in the super obese range may account for the higher prevalence rates of stroke in our study population compared with other studies.

## Limitations

Limitations of our study were the following: Psychiatric assessments of participants were done by the treating surgeon. Participants may have underreported their psychiatric symptoms, standardised psychiatric instruments were not used and the interviewer might have been biased (not objective). A small sample size was used. This study was conducted at a tertiary, academic hospital in Cape Town, and although the service receives referrals from all over the country, the findings may not be generalisable to other communities in South Africa. Finally, given the retrospective nature of the study, we relied on accurate base data entry.

Despite these limitations, the study highlights the importance of adequate psychosocial presurgical assessment and optimisation of psychiatric, medical and pain care. As part of standard practice guidelines, it is advised that a metabolic surgery program should include a dedicated multidisciplinary team (MDT).^36^ It has been shown that the presence of an MDT is an integral part of a successful metabolic surgery program, as it reduces overall mortality and morbidity in patients undergoing weight loss surgery.^37^ The APA Resource Document on Bariatric Surgery and Psychiatric Care (2016) highlights the role of psychiatrists, as part of the MDT, in the care of patients undergoing metabolic surgery,^38^ given that mental health conditions are common in metabolic surgery candidates.^14^ Pre-operative psychosocial assessment and postoperative psychosocial care aid in improving the long-term outcomes of metabolic surgery.^9,39^ Of importance are identifying factors that may impact on optimal surgical outcomes and providing recommendations to the patient and the MDT on how to manage such factors.^9^

## Conclusion

Our study showed that depressive symptoms at metabolic surgery presurgical assessment were associated with poorer QOL scores, back pain requiring non-narcotic medication and a history of stroke. This study highlights the complex interplay between metabolic, clinical and psychiatric factors in patients undergoing metabolic surgery in this setting. The study also supports the vital role of a psychiatrist as part of a MDT pre- and post-operatively in the early identification of depressive symptoms, psychoeducation and motivation of the patient to implement behaviour changes for permanent weight loss whilst adhering to the lifelong bariatric post-operatively programme. Ongoing research is required in this field, especially regarding postoperative outcomes and psychiatric comorbidities.
